# Impact of pre-pregnancy body mass index and gestational weight gain on the risk of maternal and infant pregnancy complications in Korean women

**DOI:** 10.1038/s41366-021-00946-8

**Published:** 2021-09-06

**Authors:** Hansol Choi, Joong-Yeon Lim, Nam-Kyoo Lim, Hyun Mee Ryu, Dong Wook Kwak, Jin Hoon Chung, Hee Jin Park, Hyun-Young Park

**Affiliations:** 1 Division of Population Research, Department of Precision Medicine, Korea National Institute of Health, Korea Disease Control and Prevention Agency, Cheongju, Korea; 2 Division of Healthcare Technology Development, Bureau of Advanced Health Technology Policy, Ministry of Health and Welfare, Sejong, Korea; 3 Department of Obstetrics and Gynecology, CHA Bundang Medical Center, CHA University School of Medicine, Seongnam, Korea; 4 Department of Obstetrics and Gynecology, Ajou University School of Medicine, Suwon, Korea; 5 Department of Obstetrics and Gynecology, Asan Medical Center, Ulsan University Medical School, Seoul, Korea; 6 Department of Obstetrics and Gynecology, CHA Gangnam Medical Center, CHA University School of Medicine, Seoul, Korea; 7 Department of Precision Medicine, Korea National Institute of Health, Korea Disease Control and Prevention Agency, Cheongju, Korea

**Keywords:** Epidemiology, Weight management

## Abstract

**Background/Objective:**

Healthy weight maintenance before and during pregnancy has a significant effect on pregnancy outcomes; however, there are no specific guidelines for gestational weight gain in pregnant Korean women. Therefore, we investigated the impact of pre-pregnancy body mass index (BMI) and gestational weight gain on the risk of maternal and infant pregnancy complications in pregnant Korean women.

**Methods:**

Study participants comprised 3454 singleton pregnant women from the Korean Pregnancy Outcome Study who had baseline examination and pregnancy outcome data. Maternal pre-pregnancy BMI and gestational weight gain were categorized according to the Asia-pacific regional guidelines and the Institute of Medicine recommendations, respectively. The primary outcome was any adverse outcomes, defined as the presence of one or more of the following: hypertensive disorders of pregnancy, gestational diabetes mellitus, peripartum depressive symptom, cesarean delivery, delivery complications, preterm birth, small or large weight infant, neonatal intensive care unit admission, or a congenital anomaly. Multiple logistic regression models were applied to examine the independent and combined impact of pre-pregnancy BMI and gestational weight gain on the risk of maternal and infant outcomes.

**Results:**

Obesity before pregnancy significantly increased the risk of perinatal adverse outcomes by more than 2.5 times [odds ratio (OR): 2.512, 95% confidence interval (CI): 1.817–3.473]. Compared to that in women with appropriate gestational weight gain, women with excessive weight gain had a 36.4% incremental increase in the risk of any adverse outcomes [OR: 1.364, 95% CI: 1.115–1.670]. Moreover, women who were overweight or obese before pregnancy and had excessive gestational weight gain had a three-fold increase in the risk of adverse outcomes [OR: 3.460, 95% CI: 2.210–5.417].

**Conclusion:**

This study highlights the need for appropriate weight recommendations before and during pregnancy to prevent perinatal complications in Korean women of childbearing age.

## Introduction

The increasing prevalence of obesity has become one of the most important public health concerns worldwide [1–4]. Obesity has negative effects on almost all physiological functions of the body and has a large impact on morbidity and mortality throughout the life course [1]. The prevalence of obesity in reproductive women is increasing globally, and one in four Korean women is obese [3, 4].

Overweight or obese status before pregnancy strongly influences not only metabolic complications but also adverse perinatal consequences. Higher pre-pregnancy body mass index (BMI) directly influences placental and fetal cardiometabolic development [5–8]. In addition, the offsprings of mothers with a high pre-pregnancy BMI are at increased long-term risk for obesity and cardiometabolic dysfunction [9]. Gestational weight gain is also related to the risk of maternal and infant complications [10–17]. Although gestational weight gain is necessary to ensure a healthy fetus, inappropriate weight gain is associated with adverse outcomes, including gestational diabetes mellitus (GDM), pre-eclampsia, peripartum depressive symptom, cesarean delivery, preterm birth, low birth weight, and macrosomia [10, 11, 15, 18–20].

The optimal gestational weight gain for pregnant women remains controversial. The guidelines developed by the Institute of Medicine (IOM) in 1990, and lastly revised in 2009, have become the most widely used guidelines in the world [21]. However, as the IOM guidelines are based on data from mainly Caucasian and black women in the United States (US), their generalizability may not be applicable to Asian women. Generally, Asian women are shorter and weigh less than US women do, and they have a higher risk of cardiometabolic diseases for the same age and BMI [22]. Thus, the gestational weight gain guidelines for Asian women need to be carefully considered; however, epidemiological research remains insufficient.

Appropriate weight maintenance before and during pregnancy is required to ensure both maternal and infant health. The aim of this study was to investigate the impact of pre-pregnancy BMI and gestational weight gain on the risk of maternal and infant pregnancy complications in pregnant Korean women.

## Materials and methods

### Study participants

We used data from the Korean Pregnancy Outcome Study (KPOS), which was a prospective cohort study of pregnant Korean women. A detailed description of the study protocol has been reported elsewhere [23]. During the baseline examinations conducted from 2013 to 2017, the KPOS study enrolled a total of 4195 women who visited the Cheil General Hospital and CHA Hospital for antenatal care during the first trimester. As shown in Supplementary Figure 1, after the exclusion of 111 women with twin pregnancy, miscarriage, stillbirth, misclassification, or insufficient data regarding weight during pregnancy, 3454 singleton pregnant women were included in the current analysis. Following a standard protocol, all participants completed at least one questionnaire and health examination that included a baseline assessment and had available data on the pregnancy outcomes. All participants provided written informed consent. This study was approved by the Institutional Review Boards of Cheil General Hospital (Institutional Review Board number: CGH-IRB-2013–10) and CHA Hospital (Institutional Review Board number: 2013–14-KNC13–018).

### Measurements

Self-report questionnaires on socio-demographics, medical and familial histories, health-related behaviors, psychological health, and reproductive information were completed by the participants under the supervision of trained interviewers. The delivery outcomes included the gestational age, type of labor and delivery, indication for cesarean delivery, and delivery complications. The neonatal outcomes included birth weight, Apgar score, admission to the neonatal intensive care unit, and presence of a congenital anomaly.

Anthropometric parameters were measured according to standard procedures, with participants being barefoot and wearing only light clothes. Bodyweight and standing height were measured to the nearest 0.1 kg on a digital scale and the nearest 0.1 cm on a stadiometer, respectively. The pre-pregnancy weight was measured at the first antenatal visit during the first trimester of pregnancy, and the final pregnancy weight was measured at the time of delivery. The pre-pregnancy BMI was calculated as the weight in kilograms (kg) before pregnancy, divided by the height in meters squared (m^2^), and was categorized according to the Asia-pacific regional guidelines of the World Health Organization and International Obesity Task Force as follows [24]: underweight, <18.5 kg/m^2^; normal weight, 18.5–22.9 kg/m^2^; overweight, 23.0–24.9 kg/m^2^; and obese, ≥25.0 kg/m^2^. Gestational weight gain was defined as the difference between the pre-pregnancy weight and the final weight before delivery and was classified as inadequate, appropriate, or excessive, based on the pre-pregnancy BMI and gestational weight gain, in accordance with the IOM guidelines [21]. As shown in Supplementary Table 1, the recommended gestational weight gain for pre-pregnancy BMI < 18.5 kg/m^2^, 18.5–22.9 kg/m^2^, 23.0–24.9 kg/m^2^, 25.0–29.9 kg/m^2^, and ≥30.0 kg/m^2^ were 12.5–18.0 kg, 11.5–16.0 kg, 11.5–16.0 kg, 7.0–11.5 kg, and 5.0–9.0 kg, respectively.

The primary outcome was a composite of any adverse outcomes, defined as the presence of one or more of the following: (1) hypertensive disorders of pregnancy, including gestational hypertension (systolic blood pressure ≥140 mmHg and/or diastolic blood pressure ≥90 mmHg without proteinuria after 20 weeks of gestation), preeclampsia (gestational hypertension with proteinuria ≥0.3 g in a 24-h urine collection or ≥1+ by semi-quantitative dipstick test, or end-organ dysfunction based on a platelet count <100,000/mm^3^, a creatinine level >1.1 mg/dl, elevated serum transaminase, pulmonary edema, or neurologic symptoms after 20 weeks of gestation), eclampsia (preeclampsia with seizures unrelated to other cerebral conditions), or superimposed preeclampsia (the development of symptoms of preeclampsia in a pregnant woman with chronic hypertension) [25]; (2) GDM, diagnosed using the 75-g oral glucose tolerance test with the new diagnostic criteria from the International Association of Diabetes and Pregnancy Study Group at the Cheil General Hospital, and the 100-g oral glucose tolerance test with the Carpenter-Coustan diagnostic criteria at the CHA Hospital [26, 27]; (3) peripartum depressive symptom, defined as 10 or more points on the Korean version of the Edinburgh Postnatal Depression Scale during and after pregnancy [28, 29]; (4) antenatal depressive symptom, defined as 10 or more points on the Korean version of the Edinburgh Postnatal Depression Scale during pregnancy; (5) postpartum depressive symptom, defined as 10 or more points on the Korean version of the Edinburgh Postnatal Depression Scale after pregnancy; (6) cesarean delivery; (7) delivery complications, including one or more injuries of the parturient canal, placenta abruption, premature rupture of membranes, shoulder dystocia, and uterine rupture at delivery; (8) preterm birth, defined as a gestational age at birth of less than 37 weeks; (9) small weight infant, defined as 2500 g or less at birth; (10) large weight infant, defined as 4000 g or more at birth; (11) neonatal intensive care unit admission; and (12) a congenital anomaly.

### Statistical analysis

Participants were classified according to pre-pregnancy maternal BMI and gestational weight gain for statistical analysis. Continuous variables with normal distribution were reported as means with standard deviations and were compared in general linear models. Categorical variables were reported as observed numbers with percentages and were compared using the chi-square test. Absolute risks were calculated as the percentages of women with adverse outcomes within each combination of maternal pre-pregnancy BMI and gestational weight gain categories. Multiple linear regression models were used to assess the independent associations of maternal pre-pregnancy BMI and gestational weight gain with the risk of pregnancy complications. Age, household income, educational status, marital status, parity, cigarette smoking, alcohol consumption, physical activity, history of hypertension and diabetes mellitus, and gestational age were entered into the model as covariates. Multiple logistic regression models were used to estimate odds ratios (OR) with 95% confidence intervals (CIs) for the risk of pregnancy complications according to BMI and gestational weight gain categories. An additional sensitivity analysis was conducted based on the international BMI criteria, besides the Asian classification. In addition, we repeated logistic regression analysis except for participants with a history of cesarean delivery (*n* = 432) among multiparous women from the total cesarean delivery case. All statistical analyses were performed using SAS software (version 9.4 SAS, Cary, NC, USA). Statistical significance was defined as a two-sided *p* value less than 0.05.

## Results

Among the 3454 pregnant women (mean age, 33.3 years) included in this study, 497 had underweight, 2233 had normal weight, 364 had overweight, and 360 had obese pre-pregnancy BMI status. Baseline characteristics according to the maternal pre-pregnancy BMI category are summarized in Table 1. Women who were obese before pregnancy were slightly older and had higher systolic and diastolic blood pressures, lower gestational weight gain, and higher frequencies of former and passive smoker status, hypertensive disorders of pregnancy, GDM, peripartum depressive symptom, cesarean delivery, and a large weight infant than those who had an underweight or normal weight status before pregnancy.

**Table 1:** Baseline characteristics of the study participants (*n* = 3454).

Variables	Maternal pre-pregnancy BMI					*p*
	All Participants (*n* = 3454)	Underweight, <18.5 kg/m^2^ (*n* = 497)	Normal, 18.5–22.9 kg/m^2^ (*n* = 2233)	Overweight, 23.0–24.9 kg/m^2^ (*n* = 364)	Obese, ≥25.0 kg/m^2^ (*n* = 360)	
Maternal age, years	33.3 ± 3.8	32.2 ± 3.6	33.2 ± 3.7	34.2 ± 3.5	34.6 ± 3.8	<0.001
Marital status						
Married	3387(98.1)	482(97.0)	2194 (98.3)	354 (97.3)	357 (99.2)	0.033
Unmarried	65 (1.9)	14 (2.8)	39 (1.7)	10 (2.7)	2 (0.6)	
Divorced/ Widowed/ Separated	2 (0.1)	1 (0.2)	0 (0.0)	0 (0.0)	1 (0.3)	
Educational status						
≤High school	285 (8.3)	26 (5.2)	160 (7.2)	45 (12.4)	54 (15.0)	<0.001
College or university	2582 (74.8)	395 (79.5)	1689 (75.6)	251 (69.0)	247 (68.6)	
≥Graduate school	587 (17.0)	76 (15.3)	384 (17.2)	68 (18.7)	59 (16.4)	
Household Income, million Korean-won/month						
<3	425 (12.3)	56 (11.3)	246 (11.0)	54 (14.8)	69 (19.2)	<0.001
3–5	1351 (39.1)	179 (36.0)	878 (39.3)	138 (37.9)	156 (43.3)	
≥5	1678 (48.6)	262 (52.7)	1109 (49.7)	172 (47.3)	135 (37.5)	
Pre-pregnancy BMI, kg/m^2^	21.2 ± 3.0	17.6 ± 0.7	20.5 ± 1.2	23.8 ± 0.6	27.7 ± 2.6	<0.001
Current BMI, kg/m^2^	21.6 ± 3.0	18.1 ± 0.9	21.0 ± 1.4	24.2 ± 1.0	28.0 ± 2.7	<0.001
Total gestational weight gain, kg	13.2 ± 4.7	13.3 ± 3.7	13.7 ± 4.5	12.8 ± 4.8	10.6 ± 5.6	<0.001
Gestational age, days	275.0 ± 10.0	274.9 ± 10.6	275.2 ± 9.9	275.2 ± 9.5	273.7 ± 10.5	0.0535
Blood pressure						
Systolic blood pressure, mmHg	113.8 ± 13.2	108.5 ± 11.3	112.6 ± 12.3	118.4 ± 12.9	123.8 ± 14.8	<0.001
Diastolic blood pressure, mmHg	66.2 ± 9.7	63.1 ± 9.0	65.4 ± 9.0	69.2 ± 10.0	72.4 ± 10.9	<0.001
Cigarette smoking						
Never smoked	3089 (89.4)	459 (92.4)	2000 (89.6)	326 (89.6)	304 (84.4)	0.012
Former smoker	361 (10.5)	38 (7.6)	229 (10.3)	38 (10.4)	56 (15.6)	
Current smoker	4 (0.1)	0 (0.0)	4 (0.2)	0 (0.0)	0 (0.0)	
Passive smoking	1263 (36.6)	160 (32.2)	844 (37.8)	120 (33.0)	139 (38.6)	0.041
Alcohol consumption						
Never drank	672 (19.5)	117 (23.5)	429 (19.2)	66 (18.1)	60 (16.7)	0.136
Former drinker	2778 (80.4)	380 (76.5)	1800 (80.6)	298 (81.9)	300 (83.3)	
Current drinker	4 (0.1)	0 (0.0)	4 (0.2)	0 (0.0)	0 (0.0)	
Parity	0.5 ± 0.6	0.4 ± 0.6	0.4 ± 0.6	0.6 ± 0.7	0.7 ± 0.7	<0.001
0	2052 (59.4)	320 (64.4)	1386 (62.1)	185 (50.8)	161 (44.7)	<0.001
1	1222 (35.4)	158 (31.8)	746 (33.4)	156 (42.9)	162 (45.0)	
≥2	180 (5.2)	19 (3.8)	101 (4.5)	23 (6.3)	37 (10.3)	
Type of pregnancy						
Normal	3277 (94.9)	477 (96.0)	2112 (94.6)	350 (96.2)	338 (93.9)	0.184
Ovulation induction	30 (0.9)	1 (0.2)	24 (1.1)	3 (0.8)	2 (0.6)	
Artificial insemination	33 (1.0)	4 (0.8)	25 (1.1)	3 (0.8)	1 (0.3)	
In vitro fertilization	114 (3.3)	15 (3.0)	72 (3.2)	8 (2.2)	19 (5.3)	
History of diseases						
Hypertension	17 (0.5)	0 (0.0)	5 (0.2)	2 (0.5)	10 (2.8)	<0.001
Diabetes mellitus	20 (0.6)	0 (0.0)	5 (0.2)	6 (1.6)	9 (2.5)	<0.001
Any adverse outcome	2435 (70.5)	295 (59.4)	1547 (69.3)	284 (78.0)	309 (85.8)	<0.001
Maternal	2295 (66.4)	273 (54.9)	1458 (65.3)	268 (73.6)	296 (82.2)	<0.001
Hypertensive disorders of pregnancy	42 (1.2)	3 (0.6)	18 (0.8)	5 (1.4)	16 (4.4)	<0.001
GDM	241 (7.0)	12 (2.4)	124 (5.6)	37 (10.2)	68 (18.9)	<0.001
Peripartum depressive symptom	1116 (32.3)	140 (28.2)	709 (31.8)	125 (34.3)	142 (39.4)	0.004
Antenatal depressive symptom	959 (27.8)	117 (23.5)	603 (27.0)	115 (31.6)	124 (34.4)	0.001
Postpartum depressive symptom	407 (11.8)	57 (11.5)	252 (11.3)	39 (10.7)	59 (16.4)	0.040
Cesarean delivery	1374 (39.8)	141 (28.4)	858 (38.4)	180 (49.5)	195 (54.2)	<0.001
Delivery complications	392 (11.3)	50 (10.1)	243 (10.9)	49 (13.5)	50 (13.9)	0.156
Preterm birth	167 (4.8)	24 (4.8)	102 (4.6)	17 (4.7)	24 (6.7)	0.514
Infant	601 (17.4)	79 (15.9)	369 (16.5)	77 (21.2)	76 (21.1)	0.029
Small weight infant	143 (4.1)	27 (5.4)	93 (4.2)	13 (3.6)	10 (2.8)	0.253
Large weight infant	127 (3.7)	7 (1.4)	70 (3.1)	23 (6.3)	27 (7.5)	<0.001
NICU admission	380 (11.0)	52 (10.5)	231 (10.3)	51 (14.0)	46 (12.8)	0.130
Congenital anomaly	67 (1.9)	10 (2.0)	45 (2.0)	7 (1.9)	5 (1.4)	0.884

Data expressed as means ± standard deviations or numbers (percentages).

*BMI* Body mass index, *GDM* Gestational diabetes mellitus, *NICU* Neonatal intensive care unit.

**Fig. 1:**
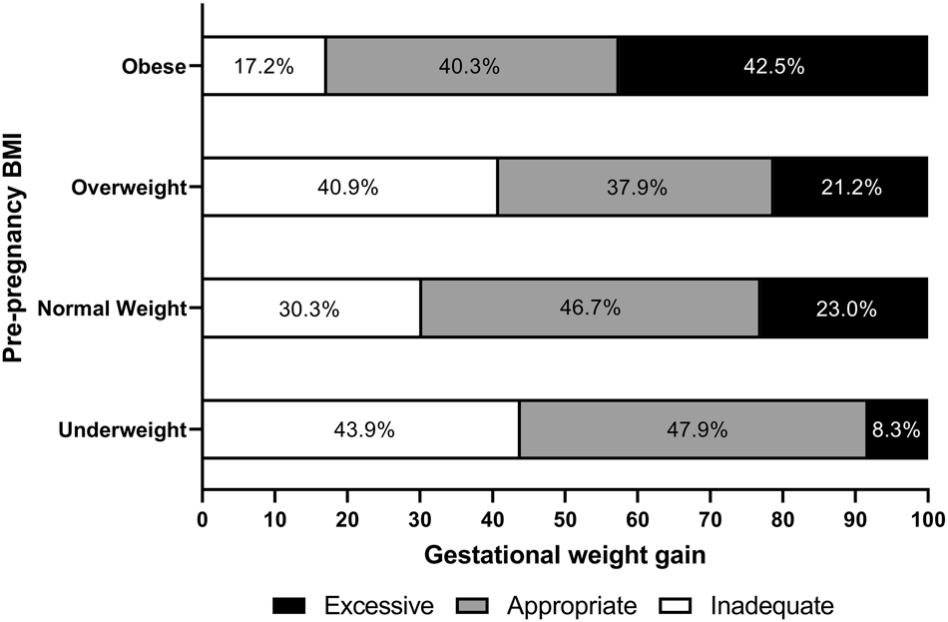
Proportions of inadequate, appropriate, or excessive gestational weight gain cases according to maternal pre-pregnancy BMI category. BMI body mass index; The proportions of inadequate, appropriate, and excessive gestational weight gain cases are shown according to the maternal pre-pregnancy BMI.

Overall, any adverse outcomes, maternal adverse outcomes, and infant adverse outcomes occurred in 70.5% (*n* = 2435), 66.4% (*n* = 2295), and 17.4% (*n* = 601) of participants, respectively. The frequency of adverse outcomes showed gradually increasing trends according to the maternal pre-pregnancy BMI categories. The proportions of inadequate, appropriate, and excessive gestational weight gain cases are shown according to the maternal pre-pregnancy BMI in Fig. 1. Although the mean gestational weight gain was the lowest in women with obesity before pregnancy, the proportion of excessive gestational weight gain cases was the highest in this group.

Associations between pre-pregnancy maternal BMI and pregnancy adverse outcomes are shown in Table 2. After adjusting for all covariates, the risk of any adverse outcomes was significantly increased in women who had overweight (OR: 1.571, 95% CI: 1.195–2.065) or obese status before pregnancy (OR: 2.512, 95% CI: 1.817–3.473) compared to that in the reference group (i.e. women who had normal weight status before pregnancy). In contrast, women who were underweight before pregnancy had a significantly reduced risk of any adverse outcomes (OR: 0.660, 95% CI: 0.536–0.813) compared to that in the reference group. Additionally, women who were obese before pregnancy had a significantly increased risk of hypertensive disorders of pregnancy (OR: 5.510, 95% CI: 2.634–11.525), GDM (OR: 3.516, 95% CI: 2.522–4.901), postpartum depressive symptom (OR: 1.388, 95% CI: 1.008–1.912), a cesarean delivery (OR: 1.749, 95% CI: 1.374–2.225), and birthing a large weight infant (OR: 2.456, 95% CI: 1.508–3.998) compared to that in the reference group. For the sensitivity analysis, we repeated the logistic regression analysis based on the international BMI criteria and found that the results were quite consistent (Supplementary Table 2).

**Table 2:** Associations between pre-pregnancy maternal BMI and adverse pregnancy outcomes (*n* = 3454).

	No. (%)	^a^Adjusted OR (95% CI) by pre-pregnancy maternal BMI			
		Underweight, <18.5 kg/m^2^ (*n* = 497)	Normal, 18.5–22.9 kg/m^2^ (*n* = 2233)	Overweight, 23.0–24.9 kg/m^2^ (*n* = 364)	Obese, ≥25.0 kg/m^2^ (*n* = 360)
Any adverse outcomes	2435 (70.5)	0.660 (0.536–0.813)	1.000	1.571 (1.195–2.065)	2.512 (1.817–3.473)
Maternal	2295 (66.4)	0.658 (0.535–0.809)	1.000	1.474 (1.137–1.911)	2.258 (1.673–3.046)
Hypertensive disorders of pregnancy	42 (1.2)	0.712 (0.203–2.498)	1.000	1.804 (0.646−5.041)	5.510 (2.634–11.525)
GDM	241 (7.0)	0.448 (0.245–0.819)	1.000	1.796 (1.216–2.653)	3.516 (2.522–4.901)
Peripartum depressive symptom	1116 (32.3)	0.884 (0.710–1.100)	1.000	1.070 (0.842–1.360)	1.217 (0.958–1.546)
Antenatal depressive symptom	959 (27.8)	0.874 (0.694–1.102)	1.000	1.173 (0.917–1.499)	1.201 (0.939–1.537)
Postpartum depressive symptom	407 (11.8)	1.103 (0.809–1.503)	1.000	0.891 (0.620–1.281)	1.388 (1.008–1.912)
Cesarean delivery	1374 (39.8)	0.654 (0.524–0.817)	1.000	1.553 (1.231–1.959)	1.749 (1.374–2.225)
Delivery complications	392 (11.3)	0.942 (0.678–1.309)	1.000	1.350 (0.962–1.892)	1.305 (0.921–1.850)
Preterm birth	167 (4.8)	0.902 (0.113–7.188)	1.000	1.580 (0.169–14.769)	1.184 (0.158–8.888)
Infant	601 (17.4)	0.930 (0.707–1.224)	1.000	1.386 (1.043–1.843)	1.247 (0.929–1.674)
Small weight infant	143 (4.1)	1.349 (0.753–2.416)	1.000	0.745 (0.342–1.621)	0.262 (0.098–0.702)
Large weight infant	127 (3.7)	0.462 (0.210–1.017)	1.000	2.001 (1.216–3.292)	2.456 (1.508–3.998)
NICU admission	380 (11.0)	0.961 (0.689–1.341)	1.000	1.486 (1.058–2.088)	1.193 (0.829–1.717)
Congenital anomaly	67 (1.9)	0.999 (0.497–2.006)	1.000	0.885 (0.389–2.012)	0.447 (0.155–1.292)

*BMI* Body mass index, *OR* Odds ratio, *CI* Confidence interval, *GDM* Gestational diabetes mellitus, *NICU* Neonatal intensive care unit.

^a^Adjusted for age, household income, educational status, marital status, parity, cigarette smoking, alcohol consumption, physical activity, history of hypertension and diabetes mellitus, and gestational age.

**Fig. 2:**
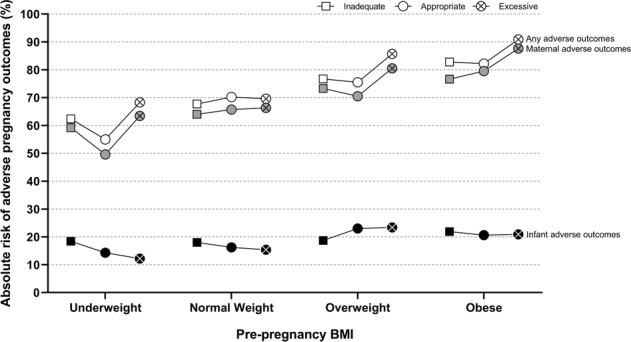
Pre-pregnancy specific absolute risks for adverse pregnancy outcomes according to gestational weight gain category. BMI body mass index; Absolute risks were calculated as the percentages of women with adverse outcomes within each combination of maternal pre-pregnancy BMI and gestational weight gain categories.

Associations between gestational weight gain and pregnancy adverse outcomes are summarized in Table 3. Compared to that in the reference group (i.e. women with appropriate gestational weight gain), women with excessive gestational weight gain had an increased risk of hypertensive disorders of pregnancy (OR: 3.253, 95% CI: 1.630–6.491), antenatal depressive symptoms (OR: 1.307, 95% CI: 1.077–1.585), a cesarean delivery (OR: 1.437, 95% CI: 1.199–1.722), and birthing a large weight infant (OR: 1.666, 95% CI: 1.116–2.487), after adjustment for all covariates. In contrast, inadequate weight gain during pregnancy was associated with an increased risk of GDM (OR: 2.160, 95% CI: 1.593–2.929) and a decreased risk of most other diseases, compared to that in the reference group. Pre-pregnancy-specific absolute risks for pregnancy adverse outcomes are shown according to the gestational weight gain category in Fig. 2. The highest absolute risk was 90.9% for excessive gestational weight gain in women with obesity before pregnancy.

**Table 3:** Associations between gestational weight gain and adverse pregnancy outcomes (*n* = 3454).

	No. (%)	^a^Adjusted OR (95% CI) by gestational weight gain		
		Inadequate	Appropriate	Excessive
Any adverse outcomes	2435 (70.5)	0.846 (0.710–1.007)	1.000	1.364 (1.115–1.670)
Maternal	2295 (66.4)	0.877 (0.739–1.040)	1.000	1.401 (1.152–1.702)
Hypertensive disorders of pregnancy	42 (1.2)	0.194 (0.059–0.635)	1.000	3.253 (1.630–6.491)
GDM	241 (7.0)	2.160 (1.593–2.929)	1.000	1.288 (0.887–1.870)
Peripartum depressive symptom	1116 (32.3)	1.115 (0.941–1.321)	1.000	1.200 (0.996–1.445)
Antenatal depressive symptom	959 (27.8)	1.175 (0.984–1.403)	1.000	1.307 (1.077–1.585)
Postpartum depressive symptom	407 (11.8)	1.176 (0.921–1.503)	1.000	1.191 (0.912–1.554)
Cesarean delivery	1374 (39.8)	0.636 (0.536–0.754)	1.000	1.437 (1.199–1.722)
Delivery complications	392 (11.3)	0.952 (0.737–1.230)	1.000	1.207 (0.923–1.576)
Preterm birth	167 (4.8)	1.793 (0.303–10.614)	1.000	0.850 (0.154–4.676)
Infant	601 (17.4)	0.955 (0.774–1.178)	1.000	1.035 (0.819–1.307)
Small weight infant	143 (4.1)	1.257 (0.789–2.003)	1.000	0.665 (0.341–1.298)
Large weight infant	127 (3.7)	0.536 (0.321–0.895)	1.000	1.666 (1.116–2.487)
NICU admission	380 (11.0)	0.982 (0.762–1.265)	1.000	0.932 (0.695–1.249)
Congenital anomaly	67 (1.9)	1.207 (0.681–2.138)	1.000	1.277 (0.687–2.377)

*OR* Odds ratio, *CI* Confidence interval, *GDM* Gestational diabetes mellitus, *NICU* Neonatal intensive care unit.

^a^Adjusted for age, household income, educational status, marital status, parity, cigarette smoking, alcohol consumption, physical activity, history of hypertension and diabetes mellitus, and gestational age.

**Fig. 3:**
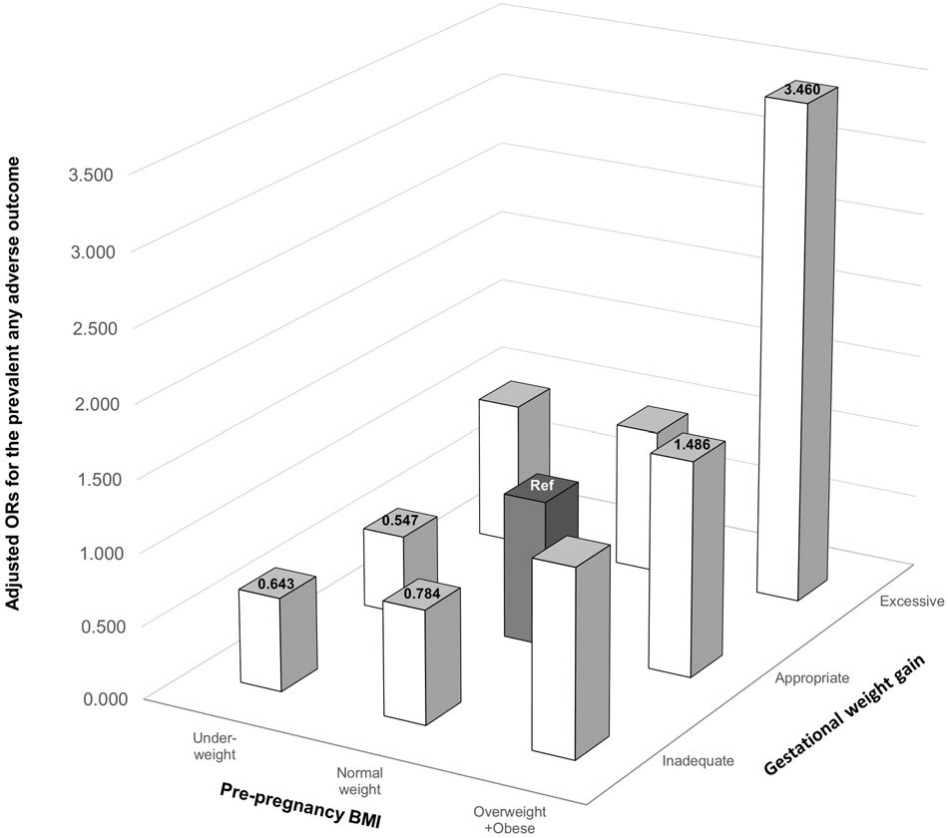
Adjusted ORs for the risk of any adverse outcomes. BMI body mass index; OR, odds ratio; Marked figures indicated statistically significant.

Fig. 3 shows the risk of pregnancy adverse outcomes according to combinations of maternal pre-pregnancy BMI and gestational weight gain categories. Women who were overweight or obese before pregnancy and had excessive weight gain during pregnancy had an increased risk for any adverse outcomes (OR: 3.460, 95% CI: 2.210–5.417) compared to the reference group (i.e., women with normal weight status before pregnancy and appropriate weight gain during pregnancy), as shown in Supplementary Table 3. Even when the gestational weight gain was appropriate, women who were overweight or obese before pregnancy had an increased risk for any adverse outcomes (OR: 1.486, 95% CI: 1.069–2.065) compared to the reference group. The risk of adverse outcomes was the lowest in women who were underweight before pregnancy and had appropriate gestational weight gain (OR: 0.547, 95% CI: 0.406–0.737). As a sensitivity analysis, we repeated the logistic regression analysis after excluding participants with a history of cesarean delivery among multiparous women and observed consistent findings in Supplementary Table 4.

## Discussion

The current study evaluated the influence of both pre-pregnancy BMI and gestational weight gain on the risk of maternal and infant pregnancy complications in pregnant Korean women. We found that higher pre-pregnancy maternal BMI and excessive gestational weight gain significantly increased the risk of pregnancy complications. Women in the highest pre-pregnancy BMI and gestational weight gain categories had approximately 2.5-times and 1.4-times, respectively, increased risk for any adverse pregnancy outcomes compared to women with normal weight status and appropriate gestational weight gain. Furthermore, the study results suggest that maternal pre-pregnancy BMI is more strongly associated with adverse pregnancy outcomes than is weight gain during pregnancy.

Unlike previous studies that studied only pre-pregnancy BMI or gestational weight gain, the present study examined the effects of both factors separately, as well as their combined effect [10, 15, 18, 19, 30, 31]. Obese before pregnancy with excessive gestational weight gain remarkably increased the risk of pregnancy complications, by three-fold, compared to the risk in women with both normal weight status before pregnancy and appropriate weight gain during pregnancy. Furthermore, women who were underweight before pregnancy and had appropriate gestational weight gain appeared to have a decreased risk, by 45%, of any adverse outcomes compared to women with normal weight status before pregnancy and appropriate weight gain during pregnancy. We also found that women who were overweight or obese before pregnancy and had inadequate gestational weight gain were not at higher risk of adverse outcomes than were other obese groups. Thus, the results suggest that pregnant women in Korea need to be offered more conservative weight recommendations.

The prevalence of overweight and obese status among women of childbearing age has risen dramatically and represents a medically important concern [2, 4]. Women with obesity tend to excessively gain weight during pregnancy, resulting in postpartum weight retention [12]. These women have not only a high risk of pregnancy complications due to their pre-pregnancy obese status, but will also have a high risk of pregnancy complications due to metabolic disorders in the future [32]. Findings from a cross-sectional study in the US indicated that, compared to women with underweight or normal weight status before pregnancy, those with overweight or obese status had an increased risk of excessive gestational weight gain [17]. Consistent with this, the present study found a significant increase in the proportion of excessive gestational weight gain cases among women with pre-pregnancy obesity compared to that among women with normal weight status. However, excessive weight gain might be more common among women with overweight or obese status because the recommended weight gain is lower and narrower in these groups [21]. In addition, smoking cessation is known to be related to excessive gestational weight gain in women with normal weight and obese status [17, 33]. Consistent with this finding, we also found that women with pre-pregnancy obesity were more likely to smoke before pregnancy, and to stop smoking when they become pregnant, despite the extremely low smoking rate among pregnant Korean women. One potential reason for this finding is that the concentration of the hormone, leptin, is significantly lower in smokers than in never smokers [34].

Interestingly, as in previous studies, we found that pre-pregnancy obesity was borderline associated with peripartum depressive symptoms, while excessive gestational weight gain was significantly associated with peripartum depressive symptoms [35, 36]. The mechanisms underlying the relationship between obesity and depression are unclear but likely involve several factors. Depression and obesity might share similar underlying pathophysiology and be manifestations of the same disease. Unhealthy behaviors, such as poor diet quality and inactivity, are commonly observed in those with overweight status, and can adversely affect mood [37, 38]. In addition, pregnant women who were overweight or obese at conception may feel stigmatized, have activity limitations imposed by carrying excess weight, and suffer from body-image dissatisfaction, leading to depression [35–38].

Our findings further support those of previous studies, including a recent systematic review and meta-analysis [11, 13, 39, 40]. As in previous studies, we found that although excessive gestational weight gain is associated with hypertensive disorders of pregnancy, antenatal depressive symptoms, cesarean delivery, and large weight infants, inadequate gestational weight gain is associated with GDM [11, 13, 39, 40]. In particular, despite the very low incidence of hypertensive disorders of pregnancy among KPOS participants, the risk of hypertensive disorders of pregnancy was significantly higher in those with excessive gestational weight gain than in those with appropriate gestational weight gain.

Similar to some previous studies, we found that inadequate gestational weight gain in obese women was associated with an increased risk of developing GDM [17, 41]. Pregnant women who have been diagnosed with GDM may receive more counseling about their glucose level and weight control and, thus, may pay more attention to diet and physical activity during the remainder of the pregnancy [17, 41]. In addition, metabolic disorders induced by GDM might influence weight gain during pregnancy.

In the present study, we used the IOM recommendations for gestational weight gain because there is no established guideline for pregnant Korean women. As the IOM guidelines are mainly based on research on Caucasians, their generalizability to other races has been a concern among Asian countries. Recent research has shown that many Asian populations are at an increased risk of cardiometabolic diseases at lower BMI levels than are non-Asians [22, 42]. In a previous Chinese retrospective study, classifying pre-pregnancy obesity as a BMI ≥ 25.0 kg/m^2^ was found to be optimal for identifying those at risk of pregnancy complications [43]. Consistent with this, we also found that BMI ≥ 25.0 kg/m^2^ is better at predicting the risk of pregnancy complications than is BMI ≥ 30.0 kg/m^2^. Since overweight and obese BMI classifications for Asian women are far lower than those for Caucasian women, the number of obese pregnant women in Korea is relatively low [1, 4]. In the present study, only 1.9% (*n* = 64) of pregnant women had a BMI of ≥30.0 kg/m^2^ before pregnancy. This suggests that gestational weight gain recommendations must be carefully considered for the prediction of the risk of pregnancy complications in Korean women.

Although the effects of weight before and after pregnancy on pregnancy complications have been previously reported, the plausible biological mechanism linking this association has not been fully elucidated. There is a direct influence of maternal obesity during pregnancy on placental and fetal cardiovascular and metabolic development. Within the cluster of cardiometabolic risk factors, obesity is one of the most important causal factors [44]. It has been shown that maternal pre-pregnancy obesity is associated with higher placental weight, placental vascular dysfunction, placental inflammation, and alterations in placental transporter and mitochondrial activity [6–8, 31, 45–47]. Even mild maternal over-nutrition induced an increased risk of adiposity and insulin intolerance in offspring in rodent models, and resulted in the up-regulation of peroxisome proliferator-activated receptor-gamma-activated genes in fetal visceral fat, with a subsequent increase in the mass of subcutaneous fat in postnatal sheep [48–52].

The present study has some limitations to consider. First, the data on pre-pregnancy body weight were mainly self-reported and assessed by recall, which may have led to a bias. However, as pre-pregnancy weight was reported during the first trimester (i.e. the recall period was relatively short), the bias effect should be small. In addition, self-reported and objective pre-pregnancy weight typically correlate well with each other, and, unless weight data are prospectively collected before pregnancy, there are no other options [53]. Second, all pregnancy complications were considered equally important; differences in the severity of outcomes were not examined. Furthermore, misclassification of the composite of any adverse outcomes may have occurred because there were some missing data for individual outcomes. However, the effects of a non-differential misclassification would have resulted in a bias toward the null hypothesis. Third, although we controlled for several potential confounders in our statistical models, there remains a possibility of residual interpregnancy confounding effects, such as dietary behaviors before and during pregnancy and gestational age, which could be directly related to pre-pregnancy BMI and gestational weight gain, and are more amenable to intervention. Fourth, we did not exclude individuals with a history of pregnancy complications, which may have influenced the results. Fifth, cesarean delivery may not be a suitable pregnancy complication since it can be caused by other influences. Finally, ethnic differences should be considered to generalize these results to other populations.

Despite these limitations, to the extent of our knowledge, this is the first study to examine the combined effects of pre-pregnancy maternal BMI and gestational weight gain on peripartum complications among pregnant Korean women in the general population. In this prospective study, pre-pregnancy obesity and excessive gestational weight gain were independently associated with maternal and infant pregnancy complications. In addition, the combination of a high BMI before pregnancy and excessive gestational weight gain can have a fatal effect in terms of adverse pregnancy outcomes. Weight control before and during pregnancy may help in reducing the risk of pregnancy complications, and gestational weight gain is a potentially modifiable risk factor for a number of adverse maternal and neonatal pregnancy complications. Especially, we recommend that women with overweight or obese status who are planning to become pregnant should reduce their BMI to within the normal range, and once pregnant, conservatively modify their gestational weight gain. Future large-scale epidemiologic studies should evaluate the optimal gestational weight gain range most suitable for pregnant Korean women, in terms of combined multiple maternal and infant characteristics that are useful for predicting pregnancy complications.

## Supplementary information

Supplemental Material

## Data Availability

All data are stored electronically in an anonymous format and are currently only available to KPOS researchers; however, data analysis collaborations may be possible through specific research proposals. Further information can be requested by e-mailing the corresponding author.

## Code availability

All programs used in this study for data analysis can be obtained from the corresponding author upon reasonable request.
