# Supplementary material for: Impact of pre-pregnancy body mass index and gestational weight gain on the risk of maternal and infant pregnancy complications in Korean women
Source: Int J Obes (Lond). 2021 Sep 6;46(1):59–67. doi: 10.1038/s41366-021-00946-8 (PMC8748202; doi:10.1038/s41366-021-00946-8)
Supplement: Supplementary file 1 — Supplemental Material [file 41366_2021_946_MOESM1_ESM.docx]

**Supplementary Material**

The study participant flow chart, definitions of the gestational weight gain and BMI categories, Associations of pre-pregnancy maternal BMI with adverse pregnancy outcomes based on the international BMI criteria, adjusted odds ratios for combinations of BMI and gestational weight gain categories, and adjusted odds ratios for combinations of BMI and gestational weight gain categories without a history of cesarean delivery are provided.

**Supplementary Table 1. Definitions of pre-pregnancy BMI and gestational weight gain categories**

| Pre-pregnancy BMI (kg/m^2^) | Total weight gain range (kg) | | |
| --- | --- | --- | --- |
|  | Inadequate | Appropriate | Excessive |
| Underweight (<18.5) | <12.5 | 12.5–18.0 | >18.0 |
| Normal (18.5–22.9) | <11.5 | 11.5–16.0 | >16.0 |
| Overweight (23.0–24.9) | <11.5 | 11.5–16.0 | >16.0 |
| Obese ^grade 1)^ (25.0–29.9) | <7.0 | 7.0–11.5 | >11.5 |
| Obese ^grade 2)^ (≥30.0) | <5.0 | 5.0–9.0 | >9.0 |

BMI, body mass index;

| **Supplementary Table 2. Associations between pre-pregnancy maternal BMI and adverse pregnancy outcomes based on the international BMI criteria (n=3454)** | | | | | | | | | | | | | | | | |
| --- | --- | --- | --- | --- | --- | --- | --- | --- | --- | --- | --- | --- | --- | --- | --- | --- |
|  | Total  No. | No. (%) | | *Adjusted OR (95% CI) by pre-pregnancy maternal BMI | | | | | | | | | | | | |
|  |  |  |  | Underweight, <18.5 kg/m^2^ (n=497) | | | | Normal, 18.5–24.9 kg/m^2^ (n=2597) | Overweight, 25.0–29.9 kg/m^2^ (n=296) | | | | Obese, ≥30.0 kg/m^2^ (n=64) | | | |
| Any adverse outcome | 3454 | 2435 | (70.5) | 0.625 | (0.508 | - | 0.768) | 1.000 | 2.017 | (1.449 | - | 2.808) | 11.006 | (2.606 | - | 46.489) |
| Maternal |  | 2295 | (66.4) | 0.627 | (0.511 | - | 0.769) | 1.000 | 1.873 | (1.375 | - | 2.551) | 6.156 | (2.154 | - | 17.592) |
| Hypertensive disorders of pregnancy |  | 42 | (1.2) | 0.646 | (0.188 | – | 2.223) | 1.000 | 3.715 | (1.672 | – | 8.254) | 11.087 | (3.941 | – | 31.197) |
| GDM |  | 241 | (7.0) | 0.404 | (0.222 | – | 0.734) | 1.000 | 2.586 | (1.808 | – | 3.698) | 6.650 | (3.715 | – | 11.901) |
| Peripartum depression |  | 1116 | (32.3) | 0.875 | (0.705 | – | 1.087) | 1.000 | 1.167 | (0.903 | – | 1.507) | 1.402 | (0.828 | – | 2.376) |
| Antenatal depression |  | 959 | (27.8) | 0.855 | (0.680 | – | 1.075) | 1.000 | 1.112 | (0.853 | – | 1.449) | 1.489 | (0.874 | – | 2.537) |
| Postpartum depression |  | 407 | (11.8) | 1.119 | (0.824 | – | 1.521) | 1.000 | 1.390 | (0.988 | – | 1.956) | 1.530 | (0.771 | – | 3.039) |
| Cesarean delivery |  | 1374 | (39.8) | 0.616 | (0.495 | - | 0.767) | 1.000 | 1.509 | (1.168 | - | 1.949) | 2.420 | (1.378 | - | 4.252) |
| Delivery complications |  | 392 | (11.3) | 0.903 | (0.653 | – | 1.249) | 1.000 | 1.145 | (0.782 | – | 1.678) | 1.697 | (0.867 | – | 3.321) |
| Preterm birth |  | 167 | (4.8) | 0.851 | (0.107 | – | 6.737) | 1.000 | 1.535 | (0.165 | – | 14.319) | 0.382 | (0.010 | – | 14.378) |
| Infant |  | 601 | (17.4) | 0.888 | (0.677 | – | 1.163) | 1.000 | 1.080 | (0.783 | – | 1.489) | 1.684 | (0.941 | – | 3.015) |
| Small weight infant |  | 143 | (4.1) | 1.394 | (0.782 | – | 2.484) | 1.000 | 0.195 | (0.058 | – | 0.658) | 0.640 | (0.135 | – | 3.036) |
| Large weight infant |  | 127 | (3.7) | 0.407 | (0.186 | – | 0.889) | 1.000 | 1.959 | (1.175 | – | 3.268) | 3.077 | (1.193 | – | 7.932) |
| NICU admission |  | 380 | (11.0) | 0.907 | (0.653 | – | 1.260) | 1.000 | 0.963 | (0.641 | – | 1.448) | 1.845 | (0.949 | – | 3.589) |
| Congenital anomaly |  | 67 | (1.9) | 1.006 | (0.504 | – | 2.007) | 1.000 | 0.297 | (0.071 | – | 1.241) | 1.081 | (0.233 | – | 5.017) |
| *Adjusted for age, household income, educational status, marital status, parity, cigarette smoking, alcohol consumption, physical activity, history of hypertension and diabetes mellitus, and gestational age  BMI, body mass index; GDM, gestational diabetes mellitus; NICU, neonatal intensive care unit; | | | | | | | | | | | | | | | | |

| **Supplementary Table 3. The combined effect of pre-pregnancy maternal BMI and gestational weight gain on adverse pregnancy outcomes (n=3454)** | | | | | | | | | | | | | | | | |
| --- | --- | --- | --- | --- | --- | --- | --- | --- | --- | --- | --- | --- | --- | --- | --- | --- |
| Adverse  outcomes | Pre-pregnancy BMI | Total  No. | No. (%) | | *Adjusted OR (95% CI) by gestational weight gain | | | | | | | | | | | |
|  |  |  |  |  | Inadequate | | | | Appropriate | | | | Excessive | | | |
| Any | Underweight | 2435 | 295 | (12.1) | 0.643 | (0.467 | - | 0.884) | 0.547 | (0.406 | - | 0.737) | 0.981 | (0.491 | - | 1.961) |
|  | Normal Weight |  | 1547 | (63.5) | 0.784 | (0.630 | - | 0.976) | 1.000 |  |  |  | 0.994 | (0.783 | - | 1.261) |
|  | Overweight + Obese |  | 593 | (24.4) | 1.289 | (0.893 | - | 1.860) | 1.486 | (1.069 | - | 2.065) | 3.460 | (2.210 | - | 5.417) |
| Maternal | Underweight | 2295 | 273 | (11.9) | 0.690 | (0.504 | - | 0.946) | 0.535 | (0.398 | - | 0.771) | 0.975 | (0.498 | - | 1.520) |
|  | Normal Weight |  | 1458 | (63.5) | 0.812 | (0.656 | - | 1.043) | 1.000 |  |  |  | 1.052 | (0.834 | - | 1.411) |
|  | Overweight + Obese |  | 564 | (24.6) | 1.235 | (0.872 | - | 1.684) | 1.449 | (1.058 | - | 2.083) | 2.960 | (1.985 | - | 3.894) |
| Infant | Underweight | 601 | 79 | (13.1) | 0.972 | (0.653 | – | 1.445) | 0.887 | (0.591 | – | 1.332) | 0.756 | (0.290 | – | 1.971) |
|  | Normal Weight |  | 369 | (61.4) | 0.968 | (0.741 | – | 1.263) | 1.000 |  |  |  | 0.983 | (0.731 | – | 1.321) |
|  | Overweight + Obese |  | 153 | (25.5) | 1.152 | (0.778 | – | 1.707) | 1.352 | (0.963 | – | 1.899) | 1.375 | (0.951 | – | 1.987) |
| *Adjusted for age, household income, educational status, marital status, parity, cigarette smoking, alcohol consumption, physical activity, history of hypertension and diabetes mellitus, and gestational age  BMI, body mass index; OR, odds ratio; CI, confidence interval; | | | | | | | | | | | | | | | | |

| **Supplementary Table 4. Combined effect of pre-pregnancy maternal BMI and gestational weight gain on adverse pregnancy outcomes after excluding a history of cesarean delivery (n=3022)** | | | | | | | | | | | | | | | | |
| --- | --- | --- | --- | --- | --- | --- | --- | --- | --- | --- | --- | --- | --- | --- | --- | --- |
| Adverse  outcomes | Pre-pregnancy BMI | Total  No. | No. (%) | | *Adjusted OR (95% CI) by gestational weight gain | | | | | | | | | | | |
|  |  |  |  |  | Inadequate | | | | Appropriate | | | | Excessive | | | |
| Any | Underweight | 2003 | 257 | (12.8) | 0.713 | (0.514 | - | 0.987) | 0.559 | (0.410 | - | 0.761) | 0.949 | (0.467 | - | 1.927) |
|  | Normal Weight |  | 1293 | (64.6) | 0.827 | (0.659 | - | 1.036) | 1.000 |  |  |  | 0.974 | (0.762 | - | 1.245) |
|  | Overweight + Obese |  | 453 | (22.6) | 1.337 | (0.910 | - | 1.964) | 1.455 | (1.031 | - | 2.053) | 3.544 | (2.239 | - | 5.609) |
| Maternal | Underweight | 1863 | 235 | (12.6) | 0.772 | (0.559 | - | 1.066) | 0.544 | (0.399 | - | 0.741) | 0.939 | (0.470 | - | 1.874) |
|  | Normal Weight |  | 1204 | (64.6) | 0.860 | (0.688 | - | 1.073) | 1.000 |  |  |  | 1.032 | (0.811 | - | 1.313) |
|  | Overweight + Obese |  | 424 | (22.8) | 1.278 | (0.884 | - | 1.848) | 1.418 | (1.018 | - | 1.975) | 3.028 | (2.005 | - | 4.574) |
| Infant | Underweight | 519 | 70 | (13.5) | 0.844 | (0.554 | - | 1.286) | 0.851 | (0.556 | - | 1.302) | 0.820 | (0.312 | - | 2.158) |
|  | Normal Weight |  | 329 | (63.4) | 0.904 | (0.681 | - | 1.200) | 1.000 |  |  |  | 0.940 | (0.686 | - | 1.288) |
|  | Overweight + Obese |  | 120 | (23.1) | 1.161 | (0.756 | - | 1.782) | 1.241 | (0.847 | - | 1.816) | 1.282 | (0.857 | - | 1.917) |
| *Adjusted for age, household income, educational status, marital status, parity, cigarette smoking, alcohol consumption, physical activity, history of hypertension and diabetes mellitus, and gestational age  BMI, body mass index; OR, odds ratio; CI, confidence interval; | | | | | | | | | | | | | | | | |

**
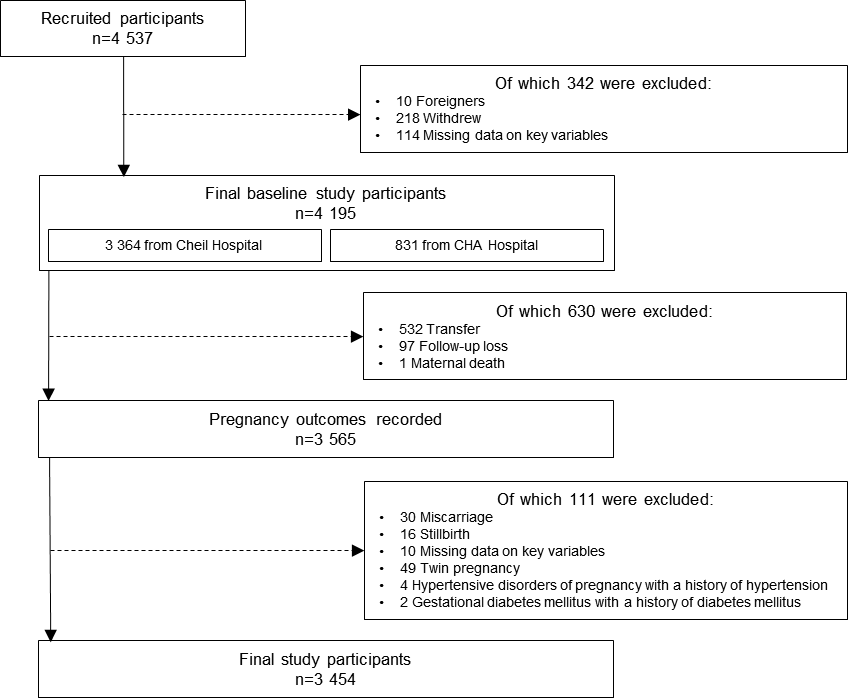
**

**Supplementary Figure 1. Flow chart of study participants**
